# Christian Associations

**Published:** 1869-01

**Authors:** 


### CHRISTIAN ASSOCIATIONS.

Young Men's Christian Associations are a new social development, and are destined, in their appropriate conduct, to work wide and worthy improvements in society; to save thousands of young men every year from falling into vicious habits and crime, disease and premature death, especially in cities and large towns; it is into these that the young men from the country crowd every year, with a view to make their fortunes; in most cases they come with high hopes and scant means; these means are often exhausted before they can find places, and then come tempters which, aided by want, destitution, and discouragement, find them an easy prey, and before they know it they are morally lost.

At other times the young clerk or apprentice gets sick, and is confined to his cheerless room in some cheap boarding-house in some out-of-the-way street, where he soon becomes lonely, dispirited and sick at heart, as well as in body; all these aggravate the disease; meanwhile it often happens that his funds become exhausted, and in such a situation it may well be conjectured that his chances of recovery are desperate.

In multitudes of other cases it happens that a young man loses his situation in business, becomes discouraged, and wanders about the street without object or aim, except with the indefinite hope that something may "turn up" to his advantage; but it oftenest happens in actual life, that while a man is patiently waiting for something to "turn up," a dozen somethings turn down.

> "For Satan finds some mischief still
> For idle hands to do;"

And that great "adversary," who is always going about seeking whom he may devour, soon finds these young men and hurries them first into bad places and practices, and incontinently into perdition.

Young men from the country are universally taught to go to meeting on the Sabbath-day, as a matter of duty, and beneficial as a mere policy; too often on coming to the city, having no acquaintances to take them to church, they soon begin to loiter around, walk the streets, go out to the park, and before they know it, they begin to like it, and the next thing we hear, they have fallen into the hands of some sharper, and that is the beginning of the end.

In another way the same result is reached; being without acquaintances, and having nothing to do at night, they get into the habit of sitting about hotels, loitering at the corners of the streets, hanging about the doors of theaters, museums, and other low forms of entertainment and excitement, and they too, are soon caught up by the designing and are made easy dupes, to fall speedily into crime and disgrace.

It is the main and fundamental design of Young Men's Christian Associations to supply a great want in all these cases. Taking the New York Young Men's Christian Association as a model and type, under the efficient presidency of W. E. Dodge, Jr., the worthy son of a noble-hearted and beneficent sire, the general plan of that is this: The city is divided into districts, a committee is appointed for each district, and different persons on this committee are appointed to different duties: to find out the young men who have recently come to town; to ascertain their object; and if it be to get into business, they strive to place them in safe, respectable and cheap boarding-houses until they can get a situation; sometimes, if found worthy, helping them to get into good places before all their money is expended; meanwhile they are taken to church on the Sabbath-day; for one or two nights a week they are encouraged to attend the meetings of the Association, where there is music, lectures, debating or other form of profitable amusement; on other nights in the week they are invited to spend an hour or two at the completely-warmed and well-lighted reading-room, provided by the philanthropy of that large-hearted man, PETER COOPER, and here, in quiet comfort, with surroundings of tidiness, cleanliness and respectability, they can spend their leisure hours in reading and reflection; or they can attend the meetings in the same building of the Polytechnic Institute or Farmers' Club, all without charge, and thus for every Sunday and for every night in the week, they are not merely kept from the dangers of the street, but are put in the way of positive good; while there is an abiding and unconscious stimulus to avoid what is wrong and to practise what is right, by the ever-present feeling that the eyes of some member of the Association may be upon them.

In another way is the young man helped to stand upright, by the friendly word of recognition given by the members as they pass him on the streets. If taken sick, committees are appointed to visit him; to see that he does not lack for medical advice, for kindly nursing, for sympathetic watchers, in all these ways is the "youth" coming to the city, hedged in from danger, and is encouraged and helped to act his part honorably; and at the same time his love for the Association grows and he becomes himself a worker for good, in the love of it, before he knows it. All honor we say to the Pioneer Young Men's Christian Association of New York city, and to young Dodge, who for some years past has done so much by his intelligence, his Christian influence, his social position, and his liberal purse to bring the Association to its present efficiency, and to the erection of its fine building on Fourth Avenue and Twenty-third Street, which will soon be finished at an expense of some two hundred thousand dollars; and thus may our country readers see that good doing commands the purses of New Yorkers; for it must be said of our merchant princes and bankers that you have only to show them that a commensurate good will be done by a specific enterprise within the bounds of the city, where they can see its operation and watch over it every day, and the money will not be long wanting, even if it requires hundreds of thousands.

And whoever has a son or brother in any town or city or country place, and has money to spare, let such be assured that the newest Young Men's Christian Association merits a donation or bequest; the living present donation being ninety-nine per cent above par; and it may be, and the chances are large, that some son or brother or lineal descendant of such a giver may be saved from moral ruin thereby. The Editor and Publisher of "Hall's Journal of Health," willing to lend a helping hand in this direction, hereby offer their magazine at half price for the year 1869, that is, twelve months' reading for seventy-five cents to all "Young Men's Christian Associations" on this continent.

